# Elderberries: A Source of Ribosome-Inactivating Proteins with Lectin Activity

**DOI:** 10.3390/molecules20022364

**Published:** 2015-01-30

**Authors:** Jesús Tejero, Pilar Jiménez, Emiliano J. Quinto, Damián Cordoba-Diaz, Manuel Garrosa, Manuel Cordoba-Diaz, Manuel J. Gayoso, Tomás Girbés

**Affiliations:** 1 Nutrición y Bromatología, Facultad de Medicina and Centro de Investigación en Nutrición, Alimentación y Dietética (CINAD), Universidad de Valladolid, Valladolid 47005, Spain; E-Mails: jesus.tejero@uva.es (J.T.); pilarj@bio.uva.es (P.J.); equinto@ped.uva.es (E.J.Q.); 2 Farmacia y Tecnología Farmacéutica, Facultad de Farmacia and Instituto Universitario de Farmacia Industrial (IUFI), Universidad Complutense de Madrid, Madrid 28040, Spain; E-Mails: damianco@farm.ucm.es (D.C.-D.); mcordoba@farm.ucm.es (M.C.-D.); 3 Biología Celular, Histología y Farmacología, Facultad de Medicina and Instituto de Neurociencias de Castilla y León (INCYL), Universidad de Valladolid, Valladolid 47005, Spain; E-Mails: garrosa@med.uva.es (M.G.); gayoso@med.uva.es (M.J.G.)

**Keywords:** *Sambucus ebulus* L., *Sambucus nigra* L., ebulin, nigrin, immunotoxins, nigrin b model

## Abstract

*Sambucus* (*Adoxaceae*) species have been used for both food and medicine purposes. Among these, *Sambucus nigra* L. (black elder), *Sambucus ebulus* L. (dwarf elder), and *Sambucus sieboldiana* L. are the most relevant species studied. Their use has been somewhat restricted due to the presence of bioactive proteins or/and low molecular weight compounds whose ingestion could trigger deleterious effects. Over the last few years, the chemical and pharmacological characteristics of *Sambucus* species have been investigated. Among the proteins present in *Sambucus* species both type 1, and type 2 ribosome-inactivating proteins (RIPs), and hololectins have been reported. The biological role played by these proteins remains unknown, although they are conjectured to be involved in defending plants against insect predators and viruses. These proteins might have an important impact on the nutritional characteristics and food safety of elderberries. Type 2 RIPs are able to interact with gut cells of insects and mammals triggering a number of specific and mostly unknown cell signals in the gut mucosa that could significantly affect animal physiology. In this paper, we describe all known RIPs that have been isolated to date from *Sambucus* species, and comment on their antiviral and entomotoxic effects, as well as their potential uses.

## 1. Introduction: Uses of *Sambucus* in Ethno-Pharmacology

Elders (*Sambucus*) belong to the family *Adoxaceae* and comprise shrubs as well as small trees found in Europe, Asia, the Americas, and Africa. Remains of seeds and charcoals of the *Sambucus* species (*S. ebulus* L., *S. nigra* L. and *S. racemosa* L.) have been used since ancient times and archaeological remains have been recovered from the Neolithic, and chalcolithic (Copper Age). They are assumed to have been used for local human subsistence in northern Italy [1] and southern France [2]. The accepted view is that wild elders were used together with other fruits to complement human cereal intake, and they have been considered to be among the most abundant fruits found at certain well-documented chalcolithic sites. To date, no data exist on elder remains in other European countries. They have, however, been used for many thousands of years in the Mediterranean area and Middle East.

Since the early use of *S. ebulus* L. and *S. nigra* L. during the Neolithic merely as food, a number of medicinal uses of these plants have been documented, with Hippocrates and Dioscorides amongst others describing them. Romans, Dacians and Gauls used them for a wide range of health purposes. Druids gave the name “Ruis” to the 13th Moon of the Celtic Year (25^th^ November–23^th^ December), and dedicated the name to the White Lady and Midsummer Solstice [3]. Owing to their medicinal properties, use of black elderberries has been documented in Europe among the most important medicinal plants. A broad variety of everyday complaints such as toothache, ear and eye problems, wounds, skin burns, colic, worms, dysentery, arthritis, rheumatism, fevers, epilepsy, and so on, have been treated over the centuries using them. 

## 2. Special Chemical Compounds Found in *Sambucus*

Like other berries, the *Sambucus* species contain a number of organic compounds that likely account for their medicinal properties [4]. These include simple phenolic acids, complex polyphenols including flavonoids, with anthocyanidins and tannins having been identified in particular. Major flavonoids present in elderberries are quercetin-3-*O*-rutinoside, quercetin-3-*O*-glucoside, kaempferol-3-*O*-rutinoside, isorhamnetin-3-*O*-rutinoside, isorhamnetin-3-*O*-glucoside, and 5-*O*-caffeoylquinic acid [5]. Two polyphenols present in *S. nigra* L. flowers, naringenin and 5-*O*-caffeoylquinic acid, increase glucose uptake and reduce fat accumulation in *Caenorhabditis elegans* [6]. Two flavonol glycosides, isorhamnetin-3-*O*-monoglycoside and quercetin-3-*O*-monoglycoside, display antiulcerogenic activity [7]. 

Among the anthocyanidins, cyanidin-3-*O*-sambubioside, cyanidin-3-sambubioside-5-glucoside, cyanidin-3-*O*-glucoside and cyanidin-3,5-diglucoside, are the most abundant anthocyanins in *S. nigra* L. and *S. ebulus* L. ripe fruits [8]. Cyanidin-3-*O*-glucoside has been found to be an anticancer compound that inhibits cell growth [9,10]. Other compounds such as the non-glycosidic iridoids 7-*O*-acetyl-patrinoside aglycone and 10-*O*-acetylpatrinoside aglycone have been isolated from the aerial parts (leaves and young branches) of *S. ebulus* L. [11], and preparations of *S. ebulus* L., rich in ursolic acid, are used to treat chronic inflammatory processes [12].

## 3. Ribosome-Inactivating Proteins (RIPs)

Ribosome-inactivating proteins (RIPs) are enzymes (E.C. 3.2.2.22) with N-glycosidase activity on the large RNA of ribosome, preventing it from engaging in protein synthesis [13,14,15]. RIPs split adenine 4324 from the 28S rRNA of the large subunit of the rat ribosome or equivalent in other eukaryotes [13,14]. This adenine is located in a loop that is involved in the interaction of the ribosome with elongation factor 2. As a result, RIPs inhibit ribosomes at the translocation step of translation [14,15]. In addition, RIPs inactivate ribosomes from certain plants [16,17] and bacteria [18]. In all these cases, the molecular mechanism of action is the same as in animal cells, namely depurination of ribosomes. Elderberry RIPs also act on DNA and polynucleotides [19,20]. 

Although the biological role played by RIPs in plants is not known [13,14,15], evidence does exist to suggest they might be involved in defending the plant against predators such as insects [21], fungi [22] and viruses [23]. RIPs may be classified into type 1 and type 2 RIPs. Additionally, there is a special group known as type 3 containing very few examples. The current paper only deals with type 1 and 2 RIPs. Type 1 RIPs such as PAP (*Phytolacca americana* antiviral protein), saporins, dianthins, beetins, *etc.*, consist of a single polypeptide chain that contains the enzymatic activity. Type 2 RIPs contain two different polypeptide chains linked by a disulphide bridge: an A chain (or active chain with enzymatic activity equivalent to a type 1 RIP) and a B chain (or binding chain able to bind to glycans of receptors present in the cell surface). The B chain enables type 2 RIPs to enter the animal cell allowing the A chain to reach the ribosomes and trigger their inactivation. Examples of such type 2 RIPs are the highly toxic ricin, abrin and other related toxins [13,14,15,21]. These type 2 RIPs are the most powerful cytotoxic agents known to date, since the IC_50_ values—concentration causing 50% inhibition—in HeLa cells are in the 1–10 pM range, and the LD_50_ values –lethal dose– in mice are in the 0.5–33.0 μg/kg body weight range.

Since 1986, when the first lectin of *S. ebulus* L. was found in rhizomes after saline extraction and further fetuin-Sepharose affinity-chromatography [24], several lectins and RIPs have been identified, isolated and characterized [13,19,25,26]. Particularly worthy of note are the type 2 RIPs from *Sambucus* species which are much less toxic to cultured animal cells and mice than ricin and are, therefore, known as non-toxic type 2 RIPs [13,14]. The reason and importance for the low intraperitoneal toxicity of type 2 RIPs as compared with ricin will be considered later.

### 3.1. Type 1 (Single Chain) Ribosome-Inactivating Proteins

*Sambucus* type 1 RIPs coexisting with type 2 RIPs have only been described to date in *S. ebulus* leaves (ebulitins) and *S. nigra* fruits (nigritins). However, this does not mean they cannot also be present in other tissues of these species and in other *Sambucus* species. Further studies are needed to reveal the biological meaning of the simultaneous presence of type 1 and type 2 in the same tissue. 

#### 3.1.1. Ebulitins α, β and γ

Ebulitins are basic proteins that belong to single-chain (type 1) RIPs and have been isolated from leaves of *S. ebulus* L. by cation-exchange chromatography, gel filtration and Blue-Sepharose affinity chromatography [27]. Three ebulitins named α, β, and γ have been isolated to date. Ebulitin α has a molecular weight of 32 kDa whereas ebulitins β and γ have an apparent Mr of 29 kDa. It has not been possible to determine their amino terminal sequence, although the amino acid composition suggests that ebulitins β and γ are similar to each other and quite different to ebulitin α. All three ebulitins inhibit protein synthesis in cell-free systems from rabbit reticulocyte lysates with an IC_50_ of 0.08, 0.34, and 1.79 nM for the α, β and γ ebulitins, whereas in the rat liver the IC_50_ values are 11.6, 10.9 and 4.8 nM, respectively. In contrast, they do not inhibit translation in plant cell-free systems. The three ebulitins display *N*-glycosidase activity on 28S rRNA rabbit reticulocyte lysates [27].

#### 3.1.2. Nigritins f1 and f2

Nigritins f1 and f2 are type 1 RIPs obtained from *S. nigra* L. fruits using the same procedure developed for ebulitins [28]. Mass spectrometry analysis has revealed that nigritins f1 and f2 have molecular masses of 24.1 kDa and 23.6 kDa, respectively. Nigritin f1 is glycosylated whereas nigritin f2 is not. Based on the structure of certain tryptic peptides obtained from these proteins, they display no amino acid N-terminal sequence homology either with one another or with other well-known RIPs. Nigritins inhibit protein synthesis in cell-free systems from rabbit reticulocyte lysates with an IC_50_ in the 3–8 nM range. In rat-liver cell-free systems, they show IC_50_ values of 15 nM for nigritin f1 and 268 nM for nigritin f2. As occurs with ebulitins, they do not inhibit translation in plant cell-free systems and display *N*-glycosidase activity on 28S rRNA rabbit reticulocyte lysates [27]. Additionally, they display topological activity, turning supercoiled circular DNA into linear and relaxed circular DNA forms [28]. 

### 3.2. Type 2 Ribosome-Inactivating Proteins from S. ebulus L.

To make easier the understanding of the different type 2 RIPs of *Sambucus* an integrative overview is presented in Table 1. 

#### 3.2.1. Ebulin l

Ebulin l is a two-chain RIP present in *S. ebulus* L leaves that was isolated by affinity chromatography on acid-treated Sepharose 6B (a dense matrix that binds proteins with affinity for d-galactose), followed by gel filtration through Superdex 75 [29]. 

Ebulin l inhibits protein synthesis in rabbit reticulocyte lysates at nearly the same concentrations as ricin with IC_50_ values of 0.15 and 0.10 nM, respectively. It also inhibits protein synthesis in cell-free systems from rat brain and rat liver (IC_50_ values of 0.09 and 0.28 nM, respectively). By contrast, it does not inhibit protein synthesis in cell-free systems from plants like *Vicia sativa, Cucumis sativus* and *Triticum aestivum*, or bacteria like *Escherichia coli* [29]. Ebulin l also displays lectin activity since it binds sugars and promotes full agglutination of human red blood cells at 51 μg/mL. Structural analyses [30] and molecular cloning of gene coding for ebulin l [31] revealed that ebulin l is composed of two subunits linked by a disulphide bridge: an A chain which evidences enzymatic activity, and a B chain which displays lectin activity. The IC_50_ of ebulin l in HeLa cells is 64.3 nM [32]. Maximum ebulin l expression is in spring shoots [33]. 

**Table 1:** Type 2 RIPs from *Sambucus*.

	Tissue	Protein	Mr (SDS-PAGE) (kDa)			Sugar Spec.	Yield ^a^	IC_50_ (nM)
			Whole	A Chain	B Chain			
**A**	leaves	ebulin l	56.0	26.0	30.0	Gal	3.2	0.15
	fruits	ebulin f	56.0	26.0	30.0	Gal	1.6	0.03
	blossoms	ebulin blo	60.0 ^b^	34.0	30.0	Gal	5.9	-
	rhizome	ebulin r1	56.0	26.0	30.0	Gal	1.34	0.34
	rhizome	ebulin r2	56.0	26.0	30.0	Gal	0.56	1.14
	rhizome	SEA I	140.0	30.0	35.0	Neu5Ac-Gal/GalNAc	6	1
**B**	bark	nigrin b	58.0	26.0	32.0	Gal/GalNAc	254	0.1
	bark	basic nigrin b	64.0	32.0	32.0	no determined	90	0.02
	bark	SNA-I	240.0	33.0	35.0	Neu5Ac-Gal/GalNAc	100	1.65
	bark	SNA-I’	120.0	32.0	35.0	Neu5Ac-Gal/GalNAc	0.2	1.25
	bark	SNRLP1	68.0	34.0	34.0	GalNAc oligomers	100 ^c^	7
	bark	SNRLP2	62.0	30.0	32.0	GalNAc oligomers	-	7
	bark	SNA-V	120.0	-	-	Gal/GalNAc	-	-
	fruits	nigrin f	58.0	26.0	31.6	Gal	1.3	0.03
	seeds	nigrin s	57.3	26.0	31.0	Gal	-	-
**C**	bark	sieboldin b	60.0	27.0	33.0	Neu5Ac-Gal/GalNAc	2.6	0.9
	bark	SSA	115.4	28.7	29.0	Neu5Ac-Gal/GalNAc	-	>100

Notes: **A**: *S. ebulus* L.; **B**: *S. nigra* L.; **C**: *S. sieboldiana* L. Type 2 RIPs have been also detected in the bark of *S. mexicana* L., *S. racemosa* L., *S. peruviana* L. and leaves of *S. nigra* L. (unpublished results). ^a^ yield per 100 g of starting material; ^b^ the molecular mass determined by mass spectrometry is 63,225; ^c^ mixture of SNRLP1 and SNLRP2. IC_50_: concentration required to inhibit translation in a 50%; unless indicated translation was assayed with reduced RIPs in rabbit reticulocyte lysates. Gal: d-galactose; Man: manose; GalNAc: *N*-acetylgalactosamine; Neu5Ac: *N*-acetyl-neuraminic acid (sialic acid) [13,26].

#### 3.2.2. Ebulins r1 and r2

Ebulins r1 and r2 were isolated from rhizomes of *S. ebulus* L. by affinity chromatography on acid-treated Sepharose 6B and gel filtration on Superdex 75, and further resolved by anion exchange chromatography on Mono-Q FPLC chromatography [34]. These proteins inhibit protein synthesis in rabbit reticulocyte lysates at lower concentrations than ebulin l (IC_50_ values of 0.05 and 0.04 nM for ebulin r1 and ebulin r2, respectively). They also inhibit protein synthesis in other mammalian cell-free systems, yet like ebulin l, do not inhibit translation in plant cell-free systems. Protein subunit structure, molecular mechanism of action, red blood cell agglutination and sugar-binding specificity are similar to those of ebulin l [29]. The ebulin r1 and r2 A chain shares the same N-terminal amino acid sequence with the ebulin l A chain, whereas it displays certain differences in the B chain. ELISA analysis shows that anti-ebulin l rabbit polyclonal antibodies react readily with ebulin l and ebulin r1 and to a less extent with ebulin r2 [34]. Both rhizome ebulins, like ricin but unlike other type 2 RIPs, act on herring sperm DNA promoting active depurination with characteristic RIP *N*-glycosidase activity but with a different efficiency [20]. 

#### 3.2.3. Ebulin f and Polyebulin f

Ebulin f was isolated from the green fruits of *S. ebulus* L. using the same procedure as for ebulin l [35,36]. Ebulin f shows five-fold more activity (IC_50_ of 0.03 nM) on protein synthesis in rabbit reticulocyte lysates than ebulin l. Activity on other cell-free systems from both mammalians and plants, and the molecular structure of ebulin f, are very similar to that of ebulin l. Yet, while the general sugar-binding specificity is the same as ebulin l, ebulin f triggers full agglutination of human red blood cells at a concentration 10 fold lower than ebulin l. In addition, ebulin f is somewhat more toxic to HeLa cells (IC_50_ of 17 nM) than ebulin l (IC_50_ value of 64.3 nM). These differences support the idea that the two ebulins are different proteins. Perhaps one of the most remarkable characteristics of ebulin f is that it accumulates in the green fruit and disappears almost completely with ripening [35]. Ebulin f is toxic to mice by intraperitoneal (i.p.) administration with an LD_50_ of 2.5 mg/kg body weight [36,37]. Ebulin f shows moderate sensitivity to pepsin as compared to the homodimeric lectin SELfd that coexists with ebulin f [35]. Both ebulin f and SELfd contain sequences present in the allergen Sam n1 found in blossoms and fruits of *S. nigra* L. [38]. Ebulin f coexists in *S. ebulus* L green fruits with a dimeric hololectin (type B-B) devoid of ribosome-inactivating activity, named SELfd [35,36]. Its polypeptide chains are identical and share very good amino acid sequence identity with the B chain of ebulin l and ebulin f and with the monomeric lectin SELm present in *S. ebulus* L. shoots. [38]. Worthy of note is that ebulin f can be polymerized with other ebulin f molecules and even with SELfd lectins to form high molecular weight aggregates which we named polyebulin and which coexist with free forms of both ebulin f and SELfd [35]. 

#### 3.2.4. Ebulin Blo

*S. ebulus* L. blossoms contain two lectins that we named ebulin blo and SELblo [25]. Ebulin blo (Mr 63,225 by mass spectrometry), like ebulin l and ebulin f, comprises two polypeptide chains of apparent Mr 28 kDa (A chain) and 35 kDa (B chain) linked by a disulphide bond. SELblo (Mr 68,432 by mass spectrometry) lacks apparent toxicity and has two identical subunits (B chains) of apparent Mr 36 kDa linked by a disulphide bond. Both proteins share amino acid sequence homology with the protein allergen Sam n1 isolated form elderberry fruits and flowers [38]. As will be shown below, ebulin blo is highly toxic to mice.

#### 3.2.5. SEA

Rhizomes of *S. ebulus* L. contain a tetrameric protein type (A-B)_2_ in which the A-B dimer is equivalent to an ebulin-type unit [19]. SEA was described years ago merely as an agglutinin specific to sialic acid, isolated by chromatography through fetuin-Sepharose [24], and no further studies were conducted on it. It has recently been found that SEA is a true type 2 RIP [19]. SEA is characterized by: (i) translation inhibitory activity in mammalian cell-free system but 100 fold less active than ricin and ebulin l; (ii) ability to release the 470 nucleotide fragment (Endo’s fragment) from rabbit reticulocyte ribosomes which is characteristic of RIPs [19]; (iii) SEA displays agglutinin activity specific to sialic acid-containing molecules. Molecular cloning indicated that SEA shares nearly 85% amino acid identity with tetrameric type 2 RIPs SNAI and SSA, and retain the Y562 residue of ricin in the 2γ-subdomain, essential for D-galactose-binding. Histological analysis revealed that SEA binds to the mucus of goblet cells of the small intestine of mice [19]. 

### 3.3. Type 2 Ribosome-Inactivating Proteins from Sambucus nigra L.

#### 3.3.1. Nigrin b (SNA-V)

Nigrin b is a type 2 RIP isolated from the bark of *S. nigra* L. [39] by the same procedure used to isolate ebulin l. For historical reasons and due to the pioneering work of the Peumans and Van Damme research group, *Sambucus* lectins are referred to as *Sambucus nigra* agglutinin (SNA) [40,41]. Accordingly, nigrin b is also known as SNA-V. The gene coding for SNA-V (nigrin b) was cloned and sequenced and displayed high amino acid sequence homology between SNA-V and ebulin l [42]. There are discrepancies in nigrin b and SNA-V quaternary structures. Depending on the purification procedure used, a dimeric A-S-S-B type structure for nigrin b [39] and a tetrameric (A-S-S-B)_2_ type structure for SNA-V [42], have been claimed. The apparent Mr of the A and B nigrin b chain are 26 kDa and 32 kDa, respectively [39]. 

Nigrin b (SNA-V) inhibits protein synthesis in mammalian cell-free systems with nearly the same efficiency as ebulin l and, also like ebulin l, is inactive on plant and bacterial ribosomes. Nigrin b (SNA-V) displays *N*-glycosidase activity with the same target ribosomal RNA as other RIPs [13]. Nigrin b (SNA-V) displays a potent *N*-glycosidase on TMV RNA, promoting multidepurination of the RNA chain which, when treated with acidic aniline, is completely degraded [43]. Nigrin b (SNA-V) promotes full agglutination of human red blood cells at 12.5 µg/mL [39].

Its IC_50_ on HeLa cells is 27.6 nM and the LD_50_ (i.p.) for mice is 12 mg/kg of body weight [32,44]. Binding to and internalization of nigrin b into HeLa cells has been studied [45]. Results initially suggest that ricin and nigrin b follow the same pathway but later differ in their routes: (i) they both bind to receptors of the plasma membrane; (ii) they are internalized, reaching an endosomic compartment; (iii) they reach a divergence point at which the pathways of the two RIPs differ substantially. From this point onwards, ricin follows a special pathway to the trans-Golgi network and then to the endoplasmic reticulum, where it is translocated to the cytosol. Nigrin b is recycled back to the plasma membrane and expelled from the cell. Passage through the endosomal pathway promotes degradation of a large number of RIP molecules (79% of ricin and 94% of nigrin b) [45]. Ricin entry into the cytosol is through translocation from the endoplasmic reticulum in a specific manner, allowing very few ricin molecules to promote cell derangement [21,45]. In contrast, nigrin entry into the cytosol seems to result from spontaneous translocation dependent on nigrin b accumulation in the endosome. This might be why only high concentrations of nigrin b are inhibitory for intact cells despite the high sensitivity of cytosolic ribosomes to nigrin b. 

#### 3.3.2. Basic Nigrin b (bNgb)

*S. nigra* L. bark contains a highly active type 2 RIP called basic nigrin b (bNgb) due to its strong basic characteristic and its capacity to bind to SP-Sepharose FF [43]. Purification was achieved by CM-Sepharose FF 0–130 mM NaCl linear gradient followed by Superdex 75, a first Mono S 0–75 mM NaCl linear gradient and second Mono S in the same conditions as the first. SDS-PAGE analysis revealed that bNgb is formed by two chains of apparent Mr 32 kDa that cannot be separated by these procedures. Mass spectrometry analysis revealed that bNgb is a heterodimer whose subunits display Mr values of 31,046 Da and 32,127 Da, and which are held together by a disulphide bridge easily reduced by dithiothreitol. Two major features of said protein are: (i) its extremely high protein synthesis inhibitory activity on rabbit reticulocyte lysates (IC_50_ 18 pg/mL) paralleled by the corresponding *N*-glycosidase activity on the 28S rRNA of rabbit reticulocyte lysates; (ii) its presence at extremely high concentrations in elderberry bark, 900 mg/kg of bark collected in September-October. Tryptic peptides obtained from bNgb indicated it is a different protein to SNA-I, SNA-I’ and SNLRP2 [43]. Nevertheless, bNgb evidences high amino acid sequence homology with SNLRPs (nearly 90%) [45]. Like nigrin b (SNA-V), bNgb binds to TMV RNA and promotes extensive depurination which, when treated with acidic aniline, undergoes complete degradation. However, and unlike nigrin b (SNA-V), bNgb binds to supercoiled pGEM4Z DNA and promotes its conversion to the relaxed-circle and linear DNA [43]. 

#### 3.3.3. Nigrin s

Nigrin s was detected in *S. nigra* L. seeds using anti-nigrin b polyclonal antibodies [46]. Although it has not been isolated, the crude extract is known to inhibit protein synthesis in rabbit reticulocyte lysates and it displays the N-glycosidase activity characteristic of common RIPs. Nigrin s is a heterodimer composed of an A chain of apparent Mr 26.3 kDa and a B chain of apparent Mr 31.0 kDa, both linked by a disulphide bridge.

#### 3.3.4. Nigrin f

Nigrin f was isolated from green and mature *S. nigra* L. fruits by affinity chromatography on acid-treated Sepharose, and anionic exchange chromatography on DEAE-Cellulose FPLC [47]. Its characteristics, such as protein synthesis inhibitory activity, human red blood cell agglutination capacity, and molecular structure, are similar to nigrin b. Nigrin f has two isoforms, a major one with an A chain of 26.3 kDa and a B chain of 31.6 kDa (most probably an isoform of nigrin s) and another with A and B chains of 28 and 30 kDa, respectively. Nigrin f is the most toxic type 2 RIP from *Sambucus* known to date. The IC_50_ value for HeLa cells is 2.9 nM (20 times more toxic than ebulin l but 2500 times less toxic than ricin). Unlike the fruits, both green and mature fruits contain nigrin f, albeit fruit ripening leads to a substantial reduction in nigrin f concentration [47]. 

#### 3.3.5. Nigrins l1 and l2

Two type 2-RIPs were found in the leaves of *S. nigra* L. (unpublished data). We have called these nigrin l1 and nigrin l2. The most salient characteristics of these proteins are that they accumulate in shoots, decay in mature leaves, and are completely absent in senescent leaves (like ebulin l). All the molecular features studied to date indicate that, although different, they might be considered the leaf forms of nigrin b.

#### 3.3.6. SNA-I

SNA-I (*Sambucus nigra* agglutinin I) was the first lectin isolated from *Sambucus*, and is the most widely studied so far. It was obtained from *S. nigra* L. bark by affinity chromatography on fetuin-agarose [48]. It is an octameric protein comprising two dissimilar subunits, A and B with an apparent Mr of 240 and an [(A-S-S-B)_2_]_2_ type structure [41]. The B chain has sugar-binding capacity and is specific for Neu5Ac(α-2,6)/Gal/GalNAc. The gene encoding SNA-I (LECSNAI) has been cloned, analysis of its sequence revealing that SNA-I is really a true type 2 RIP [49]. It contains a 1710 bp open reading frame encoding a polypeptide of 570 amino acids. In its N-terminal end, this polypeptide has a signal peptide of 28 amino acids followed by a 542 amino acid sequence that contains the A chain, the linker peptide, and the B chain. When this polypeptide is processed, proteolytic removal of the linker peptide produces a heterodimer that contains an A chain and a B chain linked by a disulphide bridge. The union of two heterodimers by another disulphide bond between the two B chains yields the tetrameric protein. It has been suggested that two tetrameric molecules could be associated by non-covalent forces into an octameric structure. SNA-I presents a 54% sequence identity with both nigrin b and nigrin f. 

SNA-I is broadly used as a powerful tool in glycoconjugate research [41], and proves particularly useful for investigating the presence of Neu5Ac(α-2,6) terminals on the surface of cancer cells [50]. In fact, induction of the corresponding sialyltranferase has been associated to the onset of several cancers [51]. In addition, it has been suggested that the intensity of staining with SNA-I might be a valid parameter for predicting recurrence of colorectal cancer [52]. A recent report demonstrates the feasibility of SNA-I for selective recognition of the STn epitope in glycoproteins with a SNA-I-biosensor [53]. 

#### 3.3.7. SNA-I’

SNA-I' was also obtained from the bark of *S. nigra* L. by affinity chromatography on fetuin-agarose and separated from SNA-I by gel filtration [54]. It is a minor component compared to SNA-I (200 times less abundant). Molecular cloning of SNA-I’ revealed the deduced amino acid sequence of SNA-I’ to be very similar to that of SNA-I and that a single cysteine residue present in the B chain of SNA-I is absent from SNA-I’, thus leading to the (A-S-S-B)_2_ type structure. SNA-I' has nearly the same poor inhibitory activity of protein synthesis. Sugar specificity has been shown to be the same as SNA-I [40,54].

#### 3.3.8. SNLRP1 and SNLRP2

SNLRPs (*Sambucus nigra* lectin-related protein) 1 and 2 are basic proteins isolated from a lectin-depleted extract from *S. nigra* L. bark using a combination of hydrophobic interaction chromatography, ion exchange chromatography and gel filtration [55]. SNLRP1 is composed of an A chain of 34 kDa and a B chain of 28 kDa, and an SNLRP2 of an A chain of 30 kDa and a B chain of 32 kDa. Both proteins inhibit protein synthesis in rabbit reticulocyte lysates although at extremely high concentrations (IC_50_ 0.5 µg/mL for a mixture of the two proteins, and IC_50_ 18 pg/mL for bNgb). 

SNLRPs 1 and 2 have also lectin activity showing carbohydrate–binding activity towards GlcNAc oligomers [41]. These proteins exhibit 91% of amino acid identity. Molecular cloning and modelling of both proteins produces a similar structure to that of ricin. Both proteins conserve a Cys that forms the disulphide bridge between the A and B chains as well as the amino acids of the active site. 

Despite the amino acid homology between SNLRPs and bNgb [55], there must be crucial differences between basic nigrin b and both SNLRPs since bNgb inhibitory activity on protein synthesis is nearly 30,000 times greater than both SNLRPs. Another important difference is that bNgb is a major protein in elder bark (0.9 g per kg of wet bark weight in samples collected in September-October) [43], while SNLRPs are minor proteins.

### 3.4. Type 2 Ribosome-Inactivating Proteins from S. sieboldiana L.

#### 3.4.1. Sieboldin b

Sieboldin b was isolated from the bark of Japanese elder (*S. sieboldiana* L.) by affinity chromatography on acid-treated Sepharose, gel filtration and cation exchange chromatography [56]. Sieboldin b inhibits protein synthesis in rabbit reticulocyte lysates at slightly lower concentrations than those of nigrin b (IC_50_ 0.015 nM), but does not inhibit it in a cell-free system obtained from *Triticum aestivum.* In addition, its molecular mechanism of action is identical to that of currently known RIPs. Sieboldin b agglutinates rabbit red blood cells at 3.1 µg/mL. 

Analysis of its carbohydrate-binding properties with a biosensor (Biacore^®^) and by ELISA indicate that sieboldin b binds to the non-reducing end galactose residues of glycoproteins and that it does not react with sialylated glycan chains. 

Inhibition of binding to galactose-bovine serum albumin by sugars indicates that, like nigrin b, sieboldin b shows a greater affinity for *N*-acetyl-d-galactosamine than for d-galactose [56]. Sieboldin b contains an A chain of 27 kDa and a B chain of 33 kDa, linked by a disulphide bridge. It shares 88% of amino acid identity with nigrin b. Its IC_50_ in HeLa cells is 12 nM (slightly lower than nigrin b) and it is not toxic via i.p. to mice up to 1.6 mg/kg body weight. The sieboldin b gene contains a 1689 bp open reading frame encoding a polypeptide of 563 amino acids. This polypeptide has a signal peptide of 25 amino acids and the sequence of 538 amino acids that, from the N-terminal end to the C-terminal end, contains the A chain, the linker peptide and the B chain. 

#### 3.4.2. SSA

SSA (*S. sieboldiana* agglutinin) was obtained from the bark of *S. sieboldiana* L. by affinity chromatography on fetuin-agarose [57]. It is a tetrameric protein composed of two dissimilar subunits of 31 and 35 kDa. It is specific for NeuAc(α-2,6)/GalNAc and for this reason was thought to be the equivalent of SNA-I in *S. nigra* L. It has recently been found that SSA, like SNA-I, is a true type 2 RIP. The A chains have N-glycosidase activity although the protein exhibits low inhibitory activity compared to other *Sambucus* RIPs. The SSA gene (LecSSA1) contains a 1710 bp open reading frame encoding a 570 amino acid polypeptide with a signal peptide of 28 amino acids, the A chain of 261 amino acids, the linker peptide of 19 amino acids, and the B chain of 261 amino acids. It conserves some amino acids that are important for structure and function, such as cysteine to form the disulphide bridge between the A and B chains, amino acids of active sites and, importantly, the amino acids involved in sugar binding. Additionally, the B chain has another cysteine that permits linking between two dimers to form a covalently stabilized tetramer.

## 4. Hololectins of *Sambucus*

*Sambucus* species contains a number of hololectins made up of polypeptide chains that share high amino acid sequence homology with the B chain of type 2 Sambucus RIPs. Several hololectins have been isolated from dwarf elder (*S. ebulus* L.). These include dimeric proteins of the B-S-S-B type such as SELld [29], SELfd [34,35], and SELblo [26], and monomeric lectins of the B type such as SEAr [33] and SEAlm [25]. All are Gal/GalNac binding lectins. 

*S. nigra* L. contains several dimeric lectins of type (B)_2_ such as SNA-II of 60 kDa [58], SNA-III of 60 kDa [59], and SNA-IV of 64 kDa [60], the three also displaying Gal/GalNAc specificity. The entomotoxic effects of the lectins will be described below. 

The Japanese elderberry (*S. sieboldiana* L.) also contains hololectins. Two monomeric lectins, SSA-b-3 and SSA-b-4, were purified from the bark with an apparent Mr of 35 and 33 kDa, respectively [61]. Molecular characterization revealed that the amino acid sequences of the encoded polypeptides were almost identical with the B-chain of a type 2 RIP of sieboldin-b, except for the absence of a cysteine residue, which is critical for heteromeric dimerization with an A-subunit [61].

## 5. Potential Biological Roles of *Sambucus* Lectins

To date there are no data on the roles played by these lectins in elderberries. Proposals therefore remain speculative. However, there is evidence to support the belief they might play a role as part of plant defence systems against viruses and insect predators. Even though SNA-I, SNA-V (nigrin b) and SNLRP are inactive on plant ribosomes, they show *in planta* polynucleotide-adenosine glycosidase activity against tobacco mosaic virus (TMV) RNA depending on the expression in high intracellular concentration [62]. 

The insecticidal activities of *Sambucus* lectins have been more widely studied. Feeding experiments with SNA-I supplemented diets have indicated lectin entomotoxic activity [63]. Available mutational analysis evidence indicates that SNA-I has insecticidal activity that depends on its B-chain carbohydrate-binding activity [64]. The occurrence of several lectins in *Sambucus*, with sugar binding specificity for Gal/GalNAc and NeuAc(α-2,6) terminals as well as with enzymatic activity, has led to the hypothesis that their joint action might exert specific signals on the carbohydrate recognition system which takes place at the cell surface [41]. 

## 6. Uses of Ribosome-Inactivating Proteins of *Sambucus* in Targeted Therapy

Over the last thirty years, type 1 and 2 RIPs have been used to construct experimental targeted drugs, in particular against cancer cells [65,66,67,68,69,70,71,72]. These drugs have been constructed chemically or genetically and contain two moieties, namely a conductor moiety that targets desired cells and a toxic moiety, usually an RIP. The chemical conjugates contain a toxin conjugated to an antibody or ligand. Genetically constructed drugs are fusion proteins containing two major kinds of domains, namely targeting domains and toxic domains. Drugs whose targeting domain derives from antibodies are known as immunotoxins. These targeted drugs have been studied *in vitro* with cultured cancer cells and have even been used in clinical trials to target different malignancies [70,71]. Ricin is the most widely used RIP in the construction of conjugates and immunotoxins for targeting cancer cells [72,73]. 

RIPs from *Sambucus*, ebulin l and nigrin b have been used in the construction of conjugates and immunotoxins. They have one crucial advantage over ricin, based on their lower toxicity (103–105 fold less toxic in cultured cells and mice than ricin). They do not therefore display unspecific toxicity at low concentration, as ricin does [74,75]. In contrast, the anti-ribosomal molecular actions of ricin, ebulin l and nigrin b are roughly the same [29,39]. The lack of toxicity of type 2 RIPs from *Sambucus* thus makes them excellent candidates as toxic moieties in the construction of immunotoxins and conjugates directed against specific targets. 

Two conjugates containing human transferrin made with *Sambucus* type 2 RIPs are transferrin-nigrin b and transferrin-ebulin l, which have been shown to be 20–40 fold more active on HeLa cells than the corresponding free RIP [76]. Since the transferrin receptor is ubiquitously expressed on normal cells and expression is increased on malignant cells, we believe these conjugates might prove useful for therapy of transferrin over expressing tumours. 

A number of immunotoxins have been constructed for antiangiogenic therapy targeting a biomarker of proliferation-dependent pathologies, such as CD105 (endoglin), a TGF-β co-receptor highly expressed in proliferating endothelial cells of the new vasculature in various cancer types [77,78,79,80,81,82]. Nigrin b and ebulin l have also been used to construct immunotoxins containing antihuman CD105 (endoglin) to target tumour neovasculature. Immunotoxins containing the mouse monoclonal antibody 44G4 raised against human CD105 as a carrier molecule, and either nigrin b [83] or ebulin l [84] have been made by covalent linking of the RIP and endoglin with SPDP. The IC_50_ for both immunotoxins were 88 pM and 150 pM for 44G4-nigrin b and 44G4-ebulin l, respectively. The specific cytotoxicity of these immunotoxins was assayed on human CD105+ cells. Both 44G4-ebulin l and 44G4-nigrin b displayed cytotoxicity with picomolar IC_50_ values. Immunofluorescence analysis indicated that 44G4-nigrin b accumulates in a perinuclear region [83]. From these data, it can be concluded that nigrin b and ebulin l can be used to construct potent and antigen-specific immunotoxins for anticancer therapy.

We constructed an immunotoxin containing both nigrin b and the monoclonal antibody MJ7 raised against mouse CD105 [85]. We studied its effect *in vivo* on mice on two murine models in which CD105 is known to be over-expressed: (i) tail injury; (ii) melanoma growing tumour. Mouse-tail injury inflicted with a needle promoted transient injury-dependent up-regulation of CD105 sufficient to trigger cytotoxicity able to damage tail tissue, leading to tail loss in some mice [85]. MJ7-nigrin b immunotoxin was tested *in vivo* in B16MEL4A5 tumour-bearing C57BL/6J mice [86]. The immunotoxin was active on B16MEL4A5 cultured cells that were shown to express CD105. For *in vivo* antitumor activity, mice were injected with enough amounts of cancer cells to trigger rapid tumour development. When the animals developed palpable solid tumours, they were subjected to treatment with the immunotoxin. Whereas neither MJ7/18 mAb nor nigrin b affected tumour development, treatment with the immunotoxin completely and steadily blocked tumour growth up to seven days, after which some tumours regrew. It is worth noting that these anti-tumour effects were exerted after one cycle of three injections in only 24 h. Histological analysis revealed that immunotoxin treated tumours present large areas of necrotic and haemorrhagic tissue. These results indicate that the MJ7-nigrin b immunotoxin attacks rapidly growing vessels of melanoma tumours once they are created, when the immunotoxin concentration is above the minimum required for cytotoxicity. This attack promotes death of endothelial cells sufficient to trigger blood haemorrhage in the growing tumour and further fibrosis in the same place. Said effect are clearly consistent with the increased expression of target CD105 in the tumour that led to local increase in CD105 density and, therefore, also increases local targeting and cytotoxicity of the immunotoxin. This contrast with other angiogenesis inhibitors that merely inhibit the appearance of new blood vessels but do not destroy them once they have appeared [87]. 

## 7. Toxicity and Histological Analysis of *Sambucus* Type 2 RIPs Administered at High Concentrations to Mice

One important difference between ricin and the *Sambucus* RIPs is toxicity to animals and cultured animal cells. Parenterally administered ricin is extremely toxic to animals [88,89,90] in contrast to *Sambucus* RIPs [13]. Differences are in the order of 103–105 fold. As indicated above, these differences afford an important advantage to *Sambucus* RIPs when used to construct immunotoxins and conjugates with far less unspecific activity than those made with ricin [83,84,85,86]. However, administering large parenteral doses of either nigrins or ebulins to mice (in the range of 2–12 mg of RIP/kg of body weight) triggers toxicity that, with the information gathered to date, seems similar to that triggered by much lower amounts of ricin. 

### 7.1. Nigrin b

**Figure 1:**
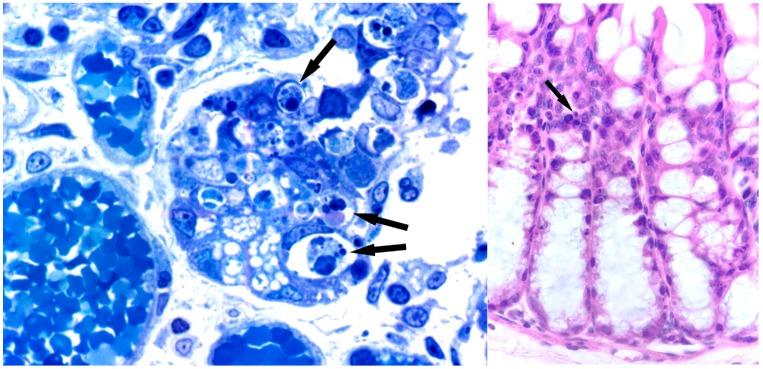
Effects of nigrin b on intestines. Histological sections of small (**left**) and large (**right**) intestine in mice six hours after injection with 16 mg/kg nigrin b. Lieberkühn’s crypts show atrophy due to increased apoptosis (arrows). (left) Semi-thin section stained with toluidine blue. ×1000; (right) Haematoxylin and eosin. ×500.

Injection through the tail vein of 16 mg/kg of body weight killed all mice studied before two days. Analysis of several major tissues by light microscopy failed to reveal gross nigrin b-promoted changes, except in intestines, which appeared highly damaged (Figure 1). As a result of the injury, the villi and crypt structures of the small intestine disappeared, leading to profuse bleeding and death. By contrast, parenteral administration (either intravenous or intraperitoneal) of sub-lethal doses of nigrin b (up to 5 mg/kg of body weight) to mice promoted reversible derangement of gut mucosa by induction of apoptosis of transit amplifying cells (TAC) of the intestinal crypts in a time-dependent course [44]. 

**Figure 2:**
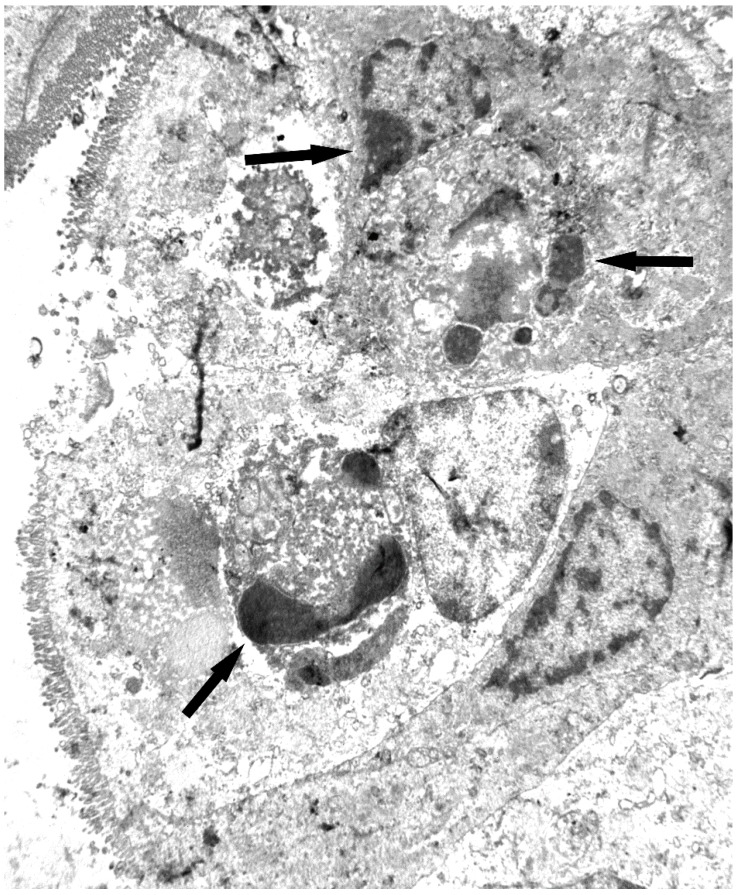
Electron micrograph of Lieberkühn’s crypt of the small intestine in a mouse 6 h after injection with 16 mg/kg nigrin b. Dead cells showing apoptotic bodies can be seen at the bottom of the crypt (arrows). ×5800.

Recent results have indicated that stem cell-derived Paneth cells are also a target of nigrin b administered at large concentrations (see Figure 2) [91]. Accordingly, despite the low unspecific toxicity exerted by nigrin b compared to ricin, parenteral injection of large amounts of nigrin b is able to kill mouse intestinal stem cells without jeopardizing the lives of the animals, thereby opening a door for its use in targeting intestinal stem cells.

Consumption of elderberry products, especially those derived from ripe fruits offers no problems of toxicity. Nevertheless, the toxic effects in mice resulting from parenteral administration of high doses of nigrin b allowed us to use said protein as a model to study nutrient absorption and toxicity [92]. One main feature of the nigrin b model is that, after 24 h of i.p. administration of sub-lethal nigrin b (10 mg/kg of body weight) there was clear body weight reduction associated to a notable increase in Evans’ blue stain accumulation in excised small intestine, an increase in myeloperoxidase activity and major derangement of intestinal crypts. Under these conditions, vitamin B_6_ accumulation in plasma from an oral bolus 24 h after nigrin b administration was reduced by nearly 50%. After 72 h nigrin b treatment, accumulation of vitamin B_6_, and small intestine crypts were almost restored although significant derangement of villi of the small intestine and large intestine crypts occurred. After eight days nigrin b treatment, all parameters had recovered with the exception of weight. The nigrin b mice model suggests that the carrier-mediated vitamin B_6_ uptake component is highly active in small intestine crypts. Said absorption model might provide insights into the processes which take place in partially altered or injured mucosa. The nigrin b model may also be used to assess the effects of proapoptopic compounds such as certain phytochemicals that promote apoptosis of altered cells, such as cancer cells. Oral administration of large doses of green tea polyphenols (GTP) and sub-lethal nigrin b separately had no effect on mouse survival [93].

However, oral administration of GTP to sub-lethal nigrin b-treated animals was found to enhance injury by nigrin b, suggesting that GTP and/or GTP-derived active metabolites might potentiate apoptosis on the damaged small intestine crypt cells. The same results were obtained when ebulin f instead of nigrin b was used (unpublished data). Since GTP action on cancer cells seems to be exerted through apoptosis, the nigrin b model would prove useful for cancer therapy research using polyphenols as driving therapeutic drugs or as an adjuvant supporting conventional therapy.

### 7.2. Ebulin f

**Figure 3:**
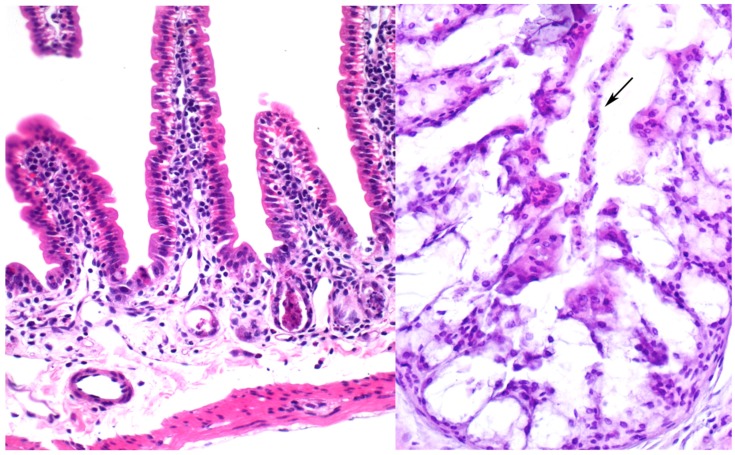
Effects of ebulin f on intestines. Histological sections of small (**left**) and large (**right**) intestine of mice stained with haematoxylin and eosin. (left) Severe atrophy of Lieberkühn’s crypts (arrows) is present in an animal injected with 5 mg/kg. ×235; (right) Derangement of the epithelium showing some dead cells sloughed off in the colonic lumen (arrow) can be seen in an animal injected with 2.8 mg/kg ebulin f. ×180.

Intraperitoneal administration (i.p.) of 5 mg/kg of body weight of ebulin f to mice killed all of them 3–5 days after administration. LD_50_ was calculated to be 2.5 mg/kg of body weight [35,36]. This is in contrast with nigrin b, which has an i.p. LD_50_ of 12 mg/kg body weight [94], and ricin with an i.p. LD_50_ of 0.023 mg/kg body weight (Figure 3) [95]. Ebulin f triggers oral toxicity with 5 mg/kg body weight and started to kill the animals three days after administration, leaving nearly 50% of the animals alive after 14 days. 

When administered orally (LD_50_: 29 mg/kg body weight) [95], ricin toxicity is lower than that shown by ebulin f. This raises the question of whether ebulin f should also be considered a poisonous toxin when administered orally, particularly because of the relatively high concentrations present in green fruits of dwarf elder. This should be taken into account since it might potentially be used in large amounts as a bioterror weapon.

*S. ebulus* L. has traditionally been considered a toxic medicinal plant that should be consumed with care, the main toxic effects occurring at the gastrointestinal level. As pointed out above, the presence of ebulin f in fruits limits its use, although it does contain large amounts of polyphenolic substances, especially fruits [96]. In order to take advantage of its antioxidants properties, a way of reducing or even eliminating toxicity thus needs to be found. We recently found that short heat treatment of dwarf elder fruit juice makes ebulin f sensitive to simulated gastric fluid without affecting the *in vitro* antioxidant and anti-free radical activities [97]. It also proved possible to eliminate the potential risks derived from the presence of lectins in dwarf elder juices without significantly reducing the content of the antioxidant compounds. 

### 7.3. Ebulin Blo

Nasal instillation of 3.75 mg/kg of body weight of an ebulin blo water solution triggers lethal toxicity in mice as little as five days after administration [26]. We believe that administration in the form of aerosol might prove even more toxic than in solution. Like ricin and ebulin f, ebulin blo toxicity raises concerns due to its potential use as a weapon by inhalation. Further studies should be carried out in order to ascertain the toxicological relevance in said context and to develop analytical procedures for detection, possible prevention and potential therapeutics. 

## 8. Entomotoxicity of *Sambucus* Proteins

At low concentrations, SNA-I and SNA-II induce caspase-dependent apoptosis in sensitive cultured midgut CF-203 cells through a mechanism that seems to be dependent on the carbohydrate-binding B chain [98]. The internalization rate of both lectins by CF-203 cells is the same and requires phosphoinositide 3-kinases [99]. These *in vitro* effects are seen also *in vivo*. SNA-I has been shown to be toxic to aphids and caterpillars [63]. SNA-II has also been shown to be entomotoxic in *Tribolium castaneum* [100]. The mechanism whereby SNA-I and SNA-II trigger toxic effects in *Tribolium castaneum* depends on their stability on gut and their passage through the peritropic matrix [100]. 

## 9. Concluding Remarks

*Sambucus* species have been used for both food and medicine. Prominent among the proteins present in *Sambucus* are Gal/GalNAc binding ribosome-inactivating proteins (RIPs) and hololectins. Little is known about their biological function, although their role in defending against insect predators and viruses has been proposed. They have an important impact on the nutritional characteristics and food safety of elderberries due to the presence of type 2 RIPs that may be toxic at high concentrations (*i.e.*, ebulin f in fruits of dwarf elder). Type 2 RIPs display lectin activity and are thus able to interact with cells of the gastrointestinal tract of insects and mammalians and trigger cellular signals in the gut mucosa that have a deep deleterious effect on animal physiology. In particular, the ebulin present in fruits (ebulin f) is toxic by oral via and therefore raises concerns vis-à-vis its potential use as a weapon. Relevant uses of these proteins are as tools in glycoconjugate and cancer research (*i.e.*, SNA-I), in targeted therapy as immunotoxins and conjugates, and as entomotoxic elements in pest control. 
